# Well-Dispersed Co/CoO/C Nanospheres with Tunable Morphology as High-Performance Anodes for Lithium Ion Batteries

**DOI:** 10.3390/ma9120955

**Published:** 2016-11-24

**Authors:** Bingqing Xu, Jingwei Li, Rujun Chen, Yuanhua Lin, Cewen Nan, Yang Shen

**Affiliations:** State Key Laboratory of New Ceramics and Fine Processing, School of Materials Science and Engineering, Tsinghua University, Beijing 100084, China; bingqingxu2013@163.com (B.X.); 18811366403@163.com (J.L.); crj12@sina.com (R.C.); cwnan@mail.tsinghua.edu.cn (C.N.); shyang_mse@mail.tsinghua.edu.cn (Y.S.)

**Keywords:** CoO anode, nanosphere, electronic conductivity, lithium ion battery

## Abstract

Well-dispersed Co/CoO/C nanospheres have been designed and constructed through a facile electrospinning method with a strategy controlling the morphology of nanocomposites via adjusting the pre-oxidized and heat treatments. Scanning electron microscopy results reveal that the as-synthesized sample pre-oxidized at 275 °C shows better spherical morphology with a diameter of around 300 nm without conspicuous agglomeration. X-ray diffraction analysis confirms the coexistence of cobalt and cobalt monoxide in the sample. Furthermore, the electrochemical tests reveal that the sample pre-oxidized at 275 °C displays excellent cycling stability with only 0.016% loss per cycle even after 400 cycles at 1000 mA·g^−1^ and enhanced high-rate capability with a specific discharge capacity of 354 mA·g^−1^ at 2000 mA·g^−1^. Besides, the sample pre-oxidized at 275 °C shows a specific capacity of 755 mA·g^−1^ at 100 mA·g^−1^ after 95 cycles. The improved electrochemical performance has been ascribed to the well dispersion of nanospheres, the improved electronic conductivity, and the structural integrity contribution from the carbon and cobalt coexisting nanocomposite. The strategy for preparing well-dispersed nanospheres by adjusting pre-oxidized and annealing processes could have insight for other oxide nanosphere synthesis.

## 1. Introduction

Nowadays, with the worsening energy crisis, lithium ion batteries (LIBs) have aroused widespread attention owing to its merits of high energy density, large voltage window, environmental friendliness, etc., when compared with other energy storage applications, such as nickel-metal hydride batteries, lead-acid batteries, supercapacitors, etc. [1,2]. Graphite has been used for commercial anodes for lithium ion batteries since the 1990s by Sony. However, low theoretical capacity (372 mA·g^−1^) and poor cycling ability are not sufficient for the development of high-capacity and high-rate lithium ion batteries in the future. Among various emerging novel anodes reported for lithium ion batteries, transition metal oxides (TMOs) have been regarded as the most appealing and competitive for their high theoretical capacity, easy manufacture, abundant raw materials, etc. SnO_2_, NiO, Co_3_O_4_, Fe_2_O_3_, and other TMOs have been studied widely both in their working mechanism and improvement methods [3,4,5].

Cobalt monoxide (CoO) has been regarded as a promising anode in lithium ion batteries because of a high theoretical capacity of 790 mA·g^−1^ (almost two times than graphite), low working potential, easy manufacture, and the saving of cobalt resources compared with Co_3_O_4_. However, low electronic conductivity and large volume expansion during cycling sets obstacles to the further utilization of CoO. Therefore, extensive endeavors have been devoted to reinforce the electronic conductivity and suppress the large volume change during lithiation and delithiation process. Generally speaking, enhancing strategies have been adopted based on the following aspects: (1) diverse nanostructures construction, such as nanospheres, nanotarrays, nanofibers, nanosheets, nanocubes, and other complex porous structures, hierarchical structures, etc. Such intricate nanostructures could provide CoO with elevated electrochemical capability on account of decreased Li ion diffusion distance and vast specific surface area which are favorable for fast Li ion transport and adequate contact between active materials and electrolyte; (2) combination with other highly electronic conductive materials, such as carbon-based materials and other transition metal oxides. Combination with carbon-based materials and nanostructures design are usually employed simultaneously [6,7,8,9,10,11,12]. In common, two methods are usually combined together, for example, multi-featured nanoboxes [13], carbon nanotube heterostructures with bead-on-string architecture [14], hierarchial nanosheets supported on amorphous carbon nanotubes [15], heterostructures attached on amorphous carbon nanotubes [16], and carbon nanotubes clad in ultrathin nanosheers [17]. In this work, well-dispersed Co/CoO/C nanospheres were prepared by a facile route inspired by adjusting the pre-oxidized temperature of electrospun Co-precursor nanofibers. Different pre-oxidized temperatures have been studied and compared in terms of final morphology of samples. The Co/CoO/C nanospheres maintain the uniform spherical shape showing a diameter distribution of about 300 nm without conspicuous agglomeration after different heat treatments. The Co/CoO/C nanospheres integrate the merits of nanospheres and their combination with metal. The in situ-generated carbon and cobalt have significant influence on achieving optimized electrochemical performance. Firstly, the in situ-generated carbon and metallic Co render the nanospheres with fast electronic diffusion rate, which favors high-rate performance. Secondly, the inactive Co and carbon can act as a mechanical framework to enhance the structural integrity and stability, suppressing the volume change during cycling, which favors for a prolonged cycle life. Apart from those merits mentioned above, metallic cobalt in nanospheres was generated in situ via facile routes without complex reduction methods, such as in a Ar-H_2_ atmosphere [18], which could bring inspiration for other transition metal oxides anode preparation. Furthermore, spherical morphology is favorable for the high tap density, having great significance for scalability and commercial production.

## 2. Experimental Section

### 2.1. Material Preparation

The Co/CoO/C nanospheres were prepared by combining electrospinning with subsequent heat treatments. Briefly, Co(Ac)_2_·4H_2_O (3 g, China National Chemicals Corporation Ltd., Beijing, China) was dissolved in the dimethyl formamide (DMF, 8 mL, China National Chemicals Corporation Ltd., Beijing, China) and stirred for 0.5 h. Then polyvinylpyrrolidone (PVP, *Mw* = 1,300,000, Alfa Corporation Ltd., Tewksbury, MA, USA) was added into the solution stirring for another 12 h to form the precursor sol. Then the sol was loaded into a syringe with a flow rate of 0.9 mL·h^−1^ under an applied electric field of 0.8 kV·cm^−1^. The as-electrospun nanofibers were then collected and pre-oxidized at 250 °C, 275 °C, and 300 °C for 0.5 h separately in air, and the three samples named as P-250, P-275, and P-300, respectively. The pre-heated powders were then carbonized at 600 °C in a N_2_ gas atmosphere. The powders were subsequently calcined for 0.5 h in air. All three samples were denoted as F-250, F-275, and F-300, respectively.

### 2.2. Characterization

X-ray structural analysis was performed by a diffractometer (Rigaku D/max-2500, Cu-Kα, 40 kV, 200 Ma, Rigaku, Tokyo, Japan). The morphology of samples was characterized with field emission scanning electron microscopy (FE-SEM; JEOL JSM-7001F, JEOL, Tokyo, Japan). The thermogravimetry-differential scanning calorimetry (TG-DSC, TGA/DSC 1, Mettler Toledo, Greifensee, Switzerland) curve was recorded at a ramping rate of 20 °C·min^−1^ from room temperature up to 700 °C under ambient atmosphere.

### 2.3. Electrochemical Tests

Electrochemical performance was tested by assembling CR2032 coin cells with prepared electrode material, lithium metal as a counter electrode, 1 M LiPF_6_ in ethyl carbonate/dimethyl carbonate with a volume ratio of 1:1 as electrolyte (SHANSHANTECH Co., Dongguan, China), and a porous polypropylene separator (Celgard 2400, Celgard Inc., Charlotte, NC, USA) in an argon-filled glove box (with O_2_ and H_2_O levels below 1 ppm). A slurry mixing 80 wt% active materials, 10 wt% super P, and 10 wt% polyvinylidene fluoride (PVDF) in *N*-methyl pyrrolidinone (NMP) solvent was stirred homogeneously for 12 h. Then the mixture was coated onto a copper foil current collector and dried at 110 °C for 12 h in −0.1 MPa vacuum. Discharge and charge tests were galvanostatically performed at room temperature in a potential range of 0.01–3 V (vs. Li/Li^+^) by a battery test system (C2001A, LAND, Wuhan, China). Cyclic voltammograms (CV) curves were obtained using a CHI760 potentiostat (Chenhua Instruments Inc., Shanghai, China) at a scan rate of 0.1 mV·s^−1^ with the voltage range of 0.01–3 V (vs. Li/Li^+^). Electrochemical impedance spectra (EIS) were performed with an amplitude of 5 mV in the range of 0.01 Hz to 100 kHz for the frequency by an impedance analyzer (IM6, Zahner Messtechnik, Kronach, Germany).

## 3. Results and Discussion

### 3.1. Structure and Morphology of the Co/CoO/C Nanospheres

**Figure 1:**
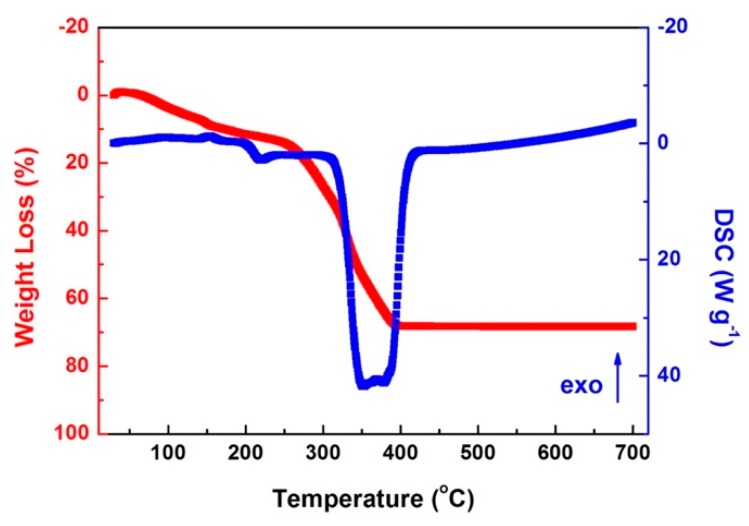
TG and DSC curves of as electrospun Co-based nanofibers.

The thermogravimetric (TG) and differential scanning calorimetry (DSC) curves of as-electrospun nanofibers are displayed in Figure 1. An evident mass loss between 280 °C and 400 °C was observed in the TG curves with an obvious endothermic peak in the DSC curves, which could be inferred as the decomposition of PVP. According to the TG and DSC results, 250 °C, 275 °C, and 300 °C were chosen to explore the influence of the pre-oxidization temperatures on the morphology of final products.

**Figure 2:**
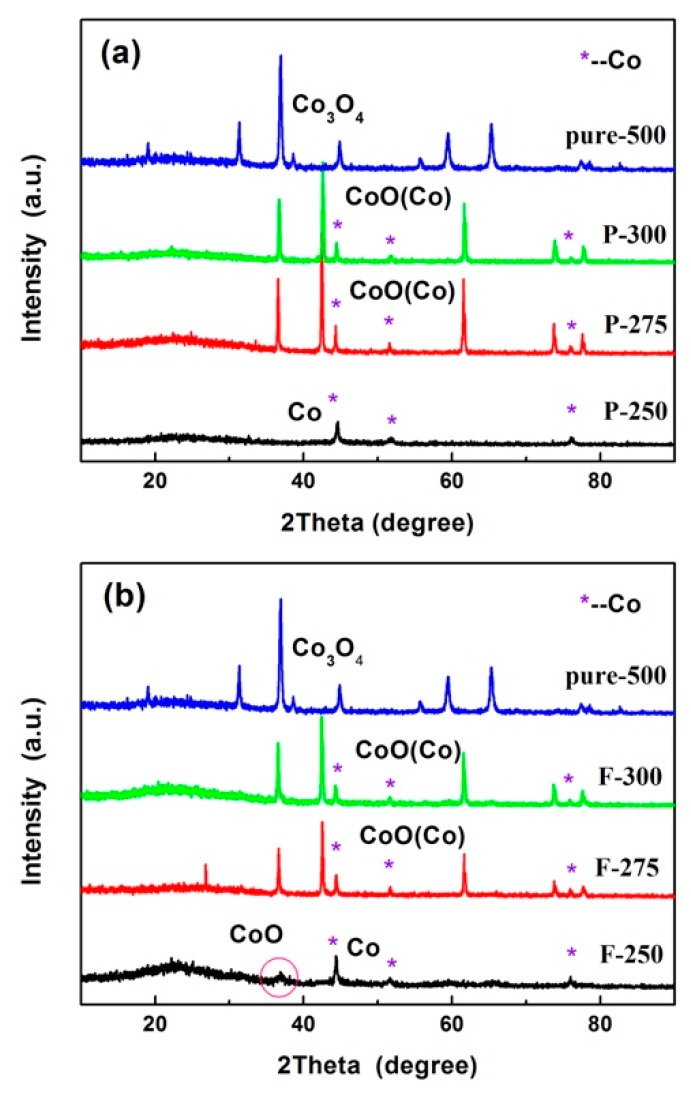
XRD patterns of (**a**) samples after pre-oxidization process and (**b**) samples after pre-oxidization, carbonization, and post-heat treatment.

The XRD patterns of three samples experiencing the pre-oxidization process and the whole heat treatments were shown in the Figure 2a,b separately. According to Figure 2a, except that the CoO phase appeared in the patterns of all three samples P-275 and P-300 (going through pre-oxidization process for 275 °C and 300 °C), Co peaks were also detected, which is different from that the pure and highly crystallized Co_3_O_4_ phase in the process of calcining in air at a mere 500 °C. However, only Co peaks appeared in the sample F-250. Furthermore, a gradual slope between 20° and 30°, which was attributed to the carbon residing in the samples. After the carbonization under N_2_ atmosphere and following post-heat treatment, the CoO and Co phase were still observed, notably, after the post-heat treatment, the CoO peaks were detected in the F-250 sample, which indicates that post-heat treatment in air helps to transfer more Co metal into the CoO phase.

**Figure 3:**
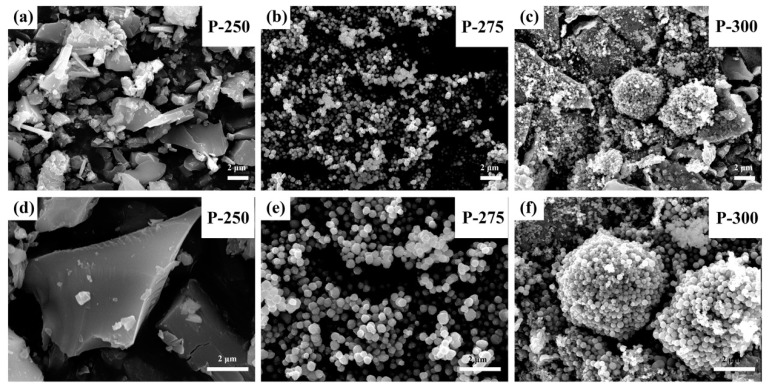
SEM images of samples after pre-oxidization at (**a**,**d**) 250 °C; (**b**,**e**) 275 °C; and (**c**,**f**) 300 °C separately.

The morphology of samples after the pre-oxidization process by scanning electron microscopy (SEM) was shown in Figure 3. From Figure 3a,d, it is clear to see a large number of flakes with a width of 4 nm and length of 6 nm in the sample P-250, which is totally different from the morphology of the as-electrospun Co-based nanofibers shown in Figure S1 (The as-electrospun nanofibers have a diameter of around 450 nm, and a continuous length of about a few micrometers with relatively smooth surface, which indicates the typical characteristics of one dimensional nanofibers). The progressive morphology with temperature changes indicate that the pre-oxidized temperature has significant influence on the morphology of products. From Figure 3, the P-275 sample shows a uniform distribution of nanofibers with a diameter at ca. 350 nm, while evident irregular microspheres or bulks assembled by nanoparticles were observed in the P-300 samples. The bulks or plates displayed in the sample P-250 reveals uncomplete decomposition of inorganic components of PVP.

**Figure 4:**
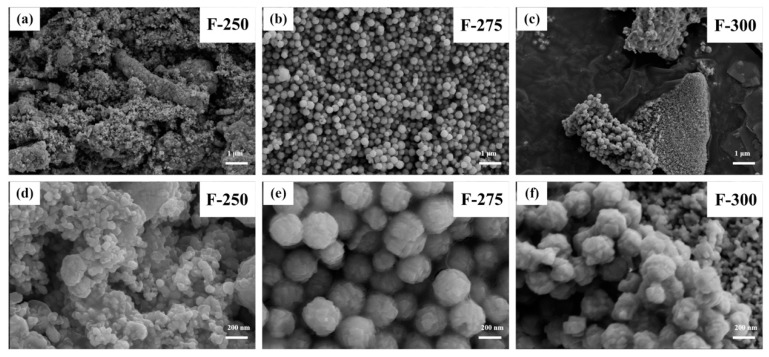
SEM images of samples after whole heat treatments with different pre-oxidization temperatures at (**a**,**d**) 250 °C; (**b**,**e**) 275 °C; and (**c**,**f**) 300 °C separately.

**Figure 5:**
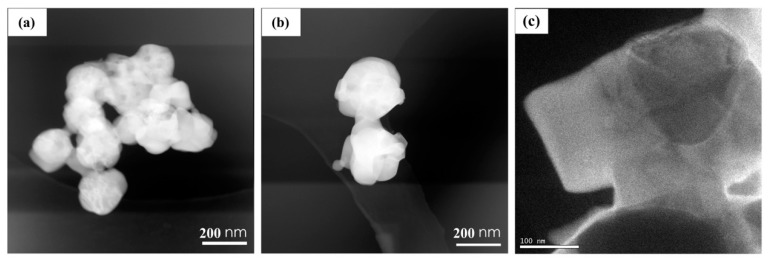

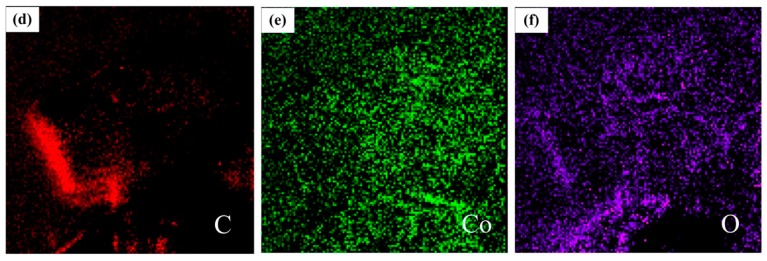
(**a**–**c**) TEM images of Co/CoO/C nanospheres (F-275); EELS mapping images of C (**d**), Co (**e**), and O (**f**) for the Co/CoO/C nanospheres (F-275).

The morphology of final products experiencing the calcination under nitrogen atmosphere and following calcination in air is shown in the Figure 4. It is apparent that F-275 products display the uniform distribution of nanospheres with a diameter of ca. 300 nm even after calcination. Comparatively, the F-300 shows the relative compactness and uniformity of nanospheres, and many nanospheres assembled into bulks instead of a mono-dispersion, as in the F-275. The F-250 sample shows the obvious agglomeration and non-uniform shapes, such as cracked bulks or micron clusters, which are unfavorable for the lithium ion diffusion. It seems that no fibrous structure was detected in the samples. From the lower resolution images for all samples in different heat treatment stages in the Figure S2, it could be seen that all three samples do not display the fibrous structures after the annealing process. This verifies that electrospun nanofibers can be smashed into particles by adjusting the annealing procedures, and rational heat treatments could turn the electrospun nanofibers into nanoparticles and nanospheres. The microstructure of the Co/CoO/C nanospheres of sample F-275 was further analyzed by TEM and EELS in Figure 5. As shown in Figure 5a,b, the TEM images display the uniform nanosphere morphology, in accordance with the SEM images. Additionally, energy-filtered TEM (EFTEM) images of single nanospheres in Figure 5c are shown in Figure 5d–f. This shows that the C element is unevenly distributed in the nanosphere, much less than Co and O. Moreover, the Co and O elements are not distributed consistently. The EFTEM results further confirm that the as-obtained Co/CoO/C nanospheres (F-275) are composed of cobalt metal, carbon, and CoO. This special structure combines the high electronic conductivity of metallic Co and carbon and the high lithium storage capacity of CoO.

### 3.2. Electrochemical Performance

**Figure 6:**
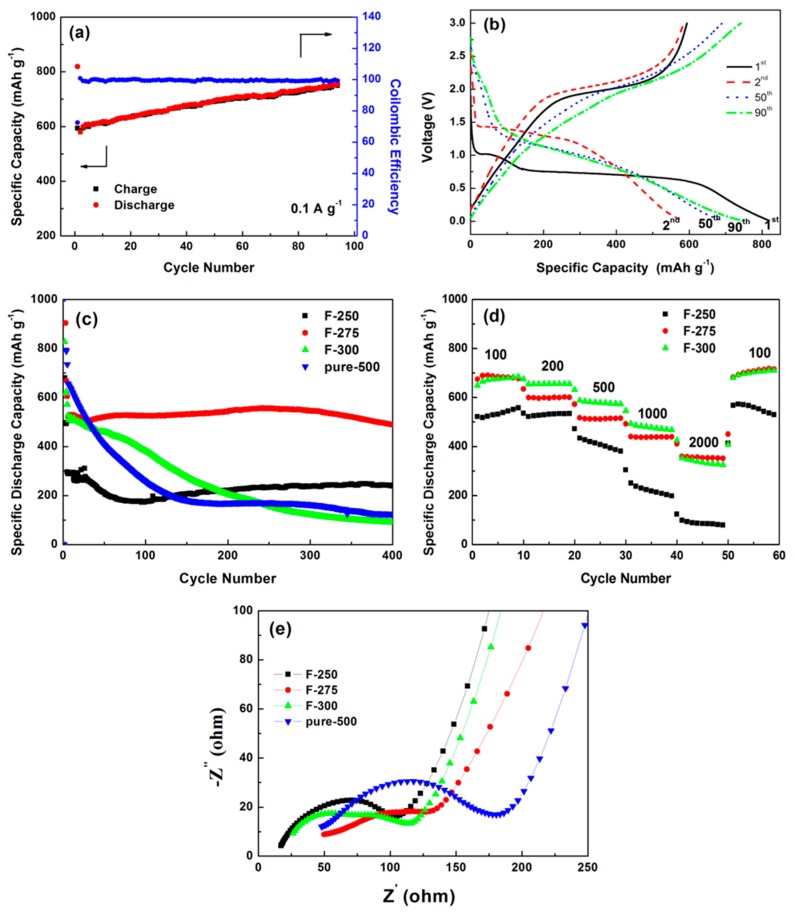
(**a**) Cycling performance of F-275 sample at 100 mA·g^−1^; (**b**) charge-discharge curves at 100 mA·g^−1^ of F-275 sample; (**c**) cycling performance of all three samples at 1000 mA·g^−1^; (**d**) rate performance of three samples at different current densities; and (**e**) electrochemical impedance spectra of three samples after rate tests.

From Figure 6a, the F-275 achieves a specific discharge capacity of around 755 mAh·g^−1^ after 95 cycles at a current density of 100 mA·g^−1^ with a coulombic efficiency of approximately 100% for every cycle. From the charge-discharge curves in Figure 6b, an obvious gradual plateau was observed at ca. 1.0 V and 0.7 V in the first discharge process, which could be ascribed to the formation of solid electrolyte interface (SEI) film and the reduction of cobalt oxides to Co [19,20,21]. The discharge and charge plateaus shifted to higher potential in the following cycles, consistent with the CV results shown in Figure S3, which indicates the reduction and oxidization process between cobalt oxides and cobalt.

Figure 6c shows the cycling performance of F-250, F-275, and F-300 samples at a current density of 1000 mA·g^−1^, respectively (the first three cycles at 100 mA·g^−1^ for the activation process). The F-275 delivers a high discharge capacity of around 523 mAh·g^−1^, and retains a stable specific discharge capacity of around 489 mAh·g^−1^ after 400 cycles, showing a capacity retention of about 93.5%, about 0.016% loss per cycle. Additionally, no apparent capacity decline was observed during 400 cycles. However, the pure Co_3_O_4_ sample shows obvious and rapid capacity decline in the initial few cycles and attains a relatively low capacity. Furthermore, F-250 and F-300 display distinct capacity loss in the initial cycles and the discharging capacities are much lower than that of F-275, which could result from the unfavorable morphology for lithium ion diffusion. The rate performance of three samples at various current densities from 100 mA·g^−1^ to 2000 mA·g^−1^ was shown in Figure 6d and that of pure Co_3_O_4_ in the Figure S4. As can be seen, the F-275 and F-300 both deliver excellent rate performance under different current densities. The F-300 displays the reversible capacity of about 675, 632, 546, 425, and 408 mAh·g^−1^ at 100, 200, 500, 1000, and 2000 mA·g^−1^ respectively, and returning to 710 mAh·g^−1^ at 100 mA·g^−1^. Additionally, the F-275 displays the reversible capacities of around 685, 600, 515, 439, and 354 mAh·g^−1^ at 100, 200, 500, 1000, and 2000 mA·g^−1^, and back to 716 mAh·g^−1^. However, the rate performance of F-250 sample shows very lower discharge capacity of only about 85 mAh·g^−1^ at 2000 A·g^−1^, and remarkable capacity decline as the increment of current densities. Pure Co_3_O_4_ delivers an inferior discharge capacity and unstable capacity retention, likewise, as shown in Figure S4. Impedance spectra of the cells after rate tests are shown in the Figure 6e. All samples contain a semicircle in the high frequency range and a sloped line in the low frequency region. The impedance data could be fitted into the equivalent electric circuit in the Figure S5. The semicircles are associated with the electrochemical reaction resistance and the slope in the low frequency refers to the solid-state diffusion of Li^+^ [22,23]. Different representations of components are illustrated in the supporting information. *R*_ct_ is a symbol of charge transfer resistance. Noticeably, *R*_ct_ of F-250, F-275, and F-300 are 87.98, 89.78, and 99.08 Ω, respectively, which are much slower than that of pure Co_3_O_4_ (142.1 Ω). Such noticeable difference displays that the Co/CoO/C nanospheres electrodes have better charge transfer capability, and the generation of cobalt and carbon could enhance the conductivity of electrodes remarkably. The F-275 displays the smallest *R*_ct_, which is consistent with the better electrochemical performance of F-275 than that of F-250 and F-300. The F-275 sample displays the most uniform nanosphere microstructure without obvious agglomeration like that in F-250 and F-300. The uniform nanospheres structure is beneficial for the electronic and lithium ions conductivity. Additionally, the microstructure of F-275 in electrodes after 500 cycles at 1000 mA·g^−1^ were further analyzed by SEM in Figure S6. It shows that spherical shapes maintain well without obvious collapse after long cycling at a high current density. Stable mechanical integrity of F-275 results in stable cycling ability with high capacity, which confirms that such structure design could enhance the electrochemical performance.

Table 1 displays the high-rate electrochemical performance comparison between this work and the literature (such as nanodisks, mesoporous nanostructures, and nanocomposites with graphene). It can be seen that the discharge and charge capacity of prepared Co/CoO/C nanospheres of F-275 in our work display a better electrochemical performance, especially in the current density of 1000 and 2000 mA·g^−1^. It shows that the unique Co/CoO/C nanosphere structure has a positive influence on enhancing the electrochemical performance of CoO electrode materials.

## 4. Conclusions

In summary, well-dispersed Co/CoO/C nanospheres were prepared successfully using a simple electrospinning process assisted with a pre-oxidization process and carbonization process. The unique Co/CoO/C nanosphere structure with generation of cobalt and carbon in situ renders the electrodes enhanced cycling stability and better rate capability when compared with the pure Co_3_O_4_ electrodes. Such remarkable enhanced electrochemical performance shows that the in-situ generation of cobalt and carbon can not only improve the electronic conductivity but also stabilize the mechanical integrity during cycling process. The facile fabrication process without any complex and demanding reduction conditions shows significance for the fabrication of other metal and metal oxide composite electrodes.

## Figures and Tables

**Table 1:** High-rate electrochemical performance comparison between this work and literature.

Sample	Preparation Method	1 A/g Capacity (mAh·g^−1^)	2 A/g Capacity (mAh·g^−1^)
Co_3_O_4_/CoO/graphene nanocomposite [24]	Sol-gel	284.22 (1.335 A·g^−1^)	163.36 (2.136 A·g^−1^)
Mesoporous CoO nanodisks [8]	Wet-chemical	~500 (0.8 A·g^−1^)	/
CoO/3D graphene composute [21]	Hydrothermal	391.2 (0.5 A·h^−1^)	/
CoO-carbon nanofiber networks [6]	Electrospinning	420	280
Carbon-Encapsulated Co_3_O_4_@CoO@Co Nanocomposites [25]	Heat treatment	~250	/
Co/CoO/C nanosphere of F-275 in this work	Electrospinning	523	354

## Supplementary Materials

The following are available online at www.mdpi.com/1996-1944/9/12/955/s1.
