# Supplementary material for: Well-Dispersed Co/CoO/C Nanospheres with Tunable Morphology as High-Performance Anodes for Lithium Ion Batteries
Source: Materials (Basel). 2016 Nov 24;9(12):955. doi: 10.3390/ma9120955 (PMC5457014; doi:10.3390/ma9120955)
Supplement: Supplementary file 1 [file materials-09-00955-s001.pdf]

Supplementary Materials: Well-Dispersed Co/CoO/C Nanospheres with Tunable Morphology as High-Performance Anodes for Lithium Ion Batteries

Bingqing Xu, Jingwei Li, Rujun Chen, Yuanhua Lin, Cewen Nan and Yang Shen

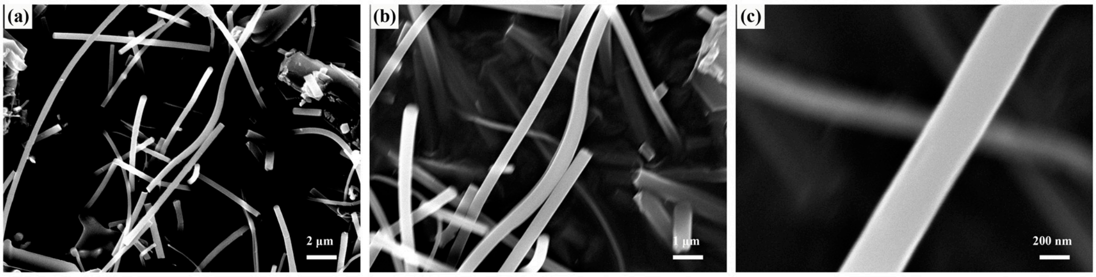

Figure S1. SEM images of as-electrospun precursor nanofibers at different resolutions: (**a**) overall morphology of fibrous structures; (**b**) fibrous structures at specific area with higher resolution and (**c**) single nanofiber structure.

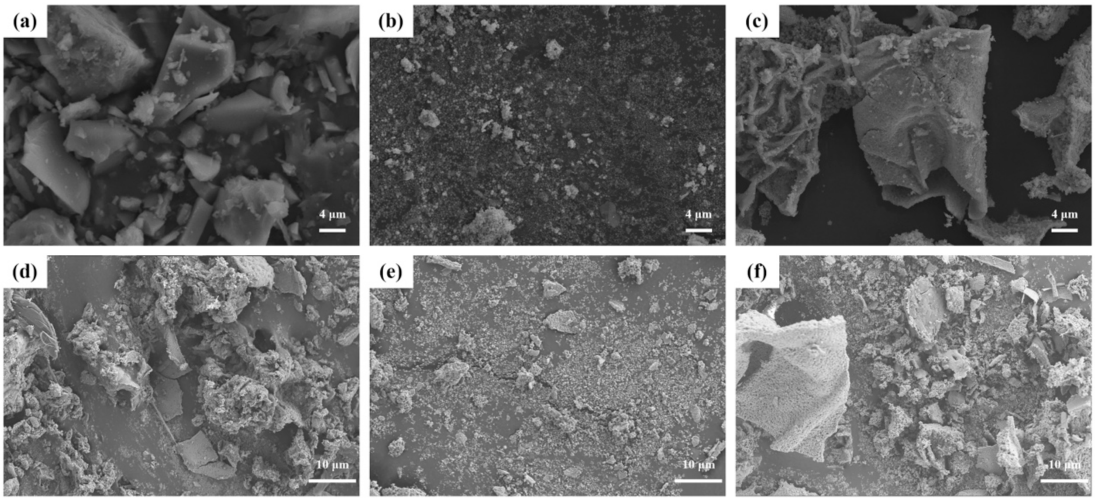

Figure S2. Low resolution SEM images of whole structure for all samples after different heat treatments stages: (**a**) P-250; (**b**) P-275; (**c**) P-300; (**d**) F-250; (**e**) F-275; (**f**) F-300.

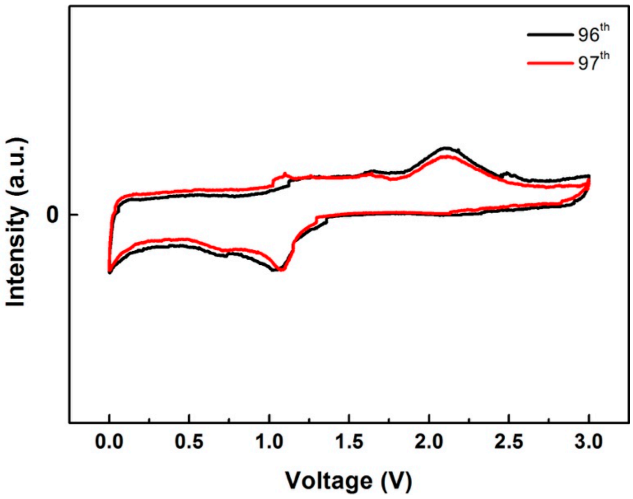

Figure S3. Cyclic voltammogram at a scanning rate of 0.1 mV·s^{-1} of F-275 sample after 95 cycles at 100 mA·g^{-1}.

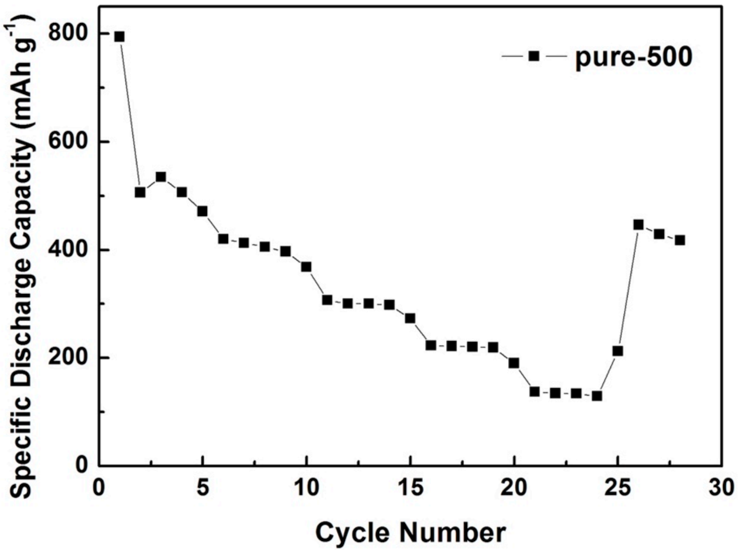

Figure S4. Rate capability of pure Co_3O_4.

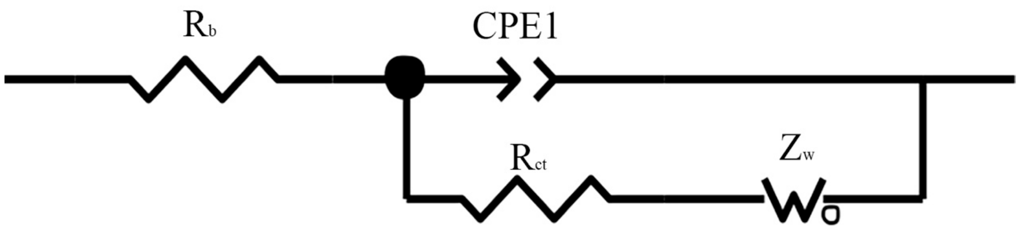

Figure S5. Equivalent electrochemical circuits. R_b: Bulk resistance, mainly reflecting the resistance of electrode and electrolyte. R_{ct}: Charge transfer resistance. CPE1: Constant phase element, related to the roughness of the partile surface. Z_w: Warburg impedance, related to the lithium ion diffusion within the particles.

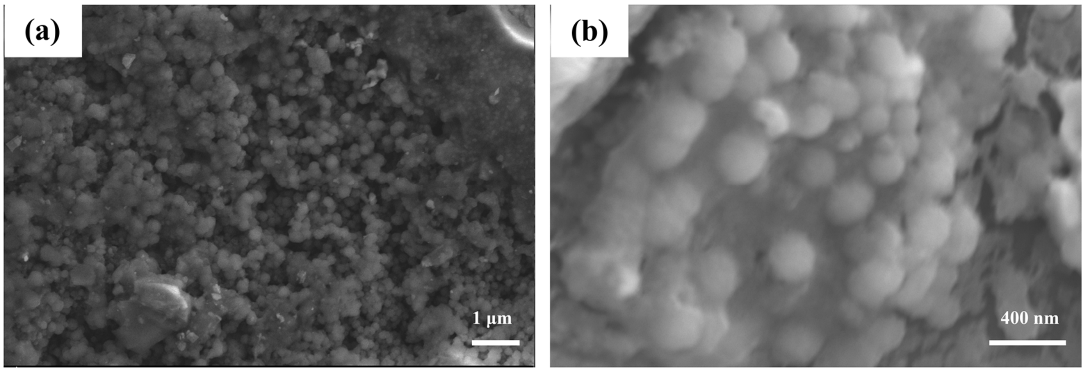

Figure S6. SEM images of electrodes with F-275 after 500 cycles at 1000 mA·g^{-1} at different resolutions. (**a**) Overall morphology of the electrodes and (**b**) specific area morphology of electrodes at higher resolution.
